# Two Distinct Training Methods for a Doctrine of Life with Healthy Heart in a Low Socioeconomic Society Model

**DOI:** 10.3390/ijerph6112883

**Published:** 2009-11-19

**Authors:** Selma Metintas, Cemalettin Kalyoncu, İnci Arıkan

**Affiliations:** Eskisehir Osmangazi University Medical Faculty, Public Health Department 26480 Meselik-Eskisehir, Turkey; E-Mails: selmamet@ogu.edu.tr (S.M.); kalyoncu@ogu.edu.tr (C.K.); iciarikan@hotmail.com (I.A.)

**Keywords:** cardiovascular risk factors, training materials, health education, community-based protection

## Abstract

This study was conducted in three stages in a semi-rural region of Eskisehir, Turkey. In the first stage, individuals selected by random sampling were evaluated for cardiovascular disease risk factors. In the second stage, Group I and Group II training materials were randomly distributed. In the third stage, the subjects were screened one year later to assess and determine if there had been any changes in their attitudes towards the dangers of cardiovascular diseases. The number of active smokers significantly decreased after the training in the both groups. The percentage of people with regulated blood pressure exhibited an increase in Group II more than Group I.

## 1. Introduction

Cardiovascular disease is a very common public health issue throughout the world. Despite the decrease in cardiovascular-related disease incidence and death rates in developed countries, an increase in these indicators is being observed in developing countries due to demographic transformations and changes in lifestyle [1–4].

Turkey is one of several countries with a high rate of cardiovascular disease prevalence. Cardiovascular disease ranks highly among all death causes in both females and males, and the number of such deaths is expected to increase 1.8-fold in females and 2.3-fold in males by 2030 [5]. Not surprisingly, given the high prevalence of cardiovascular disease, Turkey also has a high prevalence of cardiovascular risk factors: smoking prevalence among individuals above the age of 18 is 49.4% in males and 20% in females; sedentary lifestyle prevalence is 15.9% among males and 23.6% among females; body mass index (BMI) > 30 is 9.7% and 14.5% for males and females, respectively [6,7]; and hypertension prevalence is 27.5% in males, but reportedly 36.1% in females among the population over 20 years of age [8].

The trend of decreasing incidence of cardiovascular disease in developed countries is largely due to the impact of society-based protection programs [9,10]. However, financial spending on programs aiming to keep cardiovascular disease under control is far lower in developing countries than in developed countries. In order to keep cardiovascular diseases under control, a salutogenic approach to developing efficient and cheap control methods is needed to decrease the risk factors [11].

A large number of cardiovascular disease intervention programs were designed to evaluate the possible effectiveness of society-based intervention [9,10] to cause change in cardiovascular disease knowledge and risk-factors. These programs have used mass media health education programs, radio, television, printed media, risk factor screening, adult education classes, community-wide risk factor education campaigns at al. The programs evaluated the effectiveness of society-based interventions to modify cardiovascular disease knowledge and risk factors in developed country. However, little research has been performed on developing countries.

Another issue in developing countries is the widespread inequality in access to health care services. In order to diminish this inequality, for determining which groups have difficulty accessing health care services and in presenting relevant solutions preference should be given to groups with low socio-economic levels.

This study aims to compare two training material formats in order to analyze the effect of distributing information about healthy heart life principles on behavioral changes in a population with a low socio-economic level.

## 2. Methods

### 2.1. Study Population

The study was conducted in a geographic areas with semi-rural characteristics, located in the Central Anatolia Region of Eskisehir. Permission for this investigation was granted by the Osmangazi University Medical Faculty Ethical Committee (Study number of Ethical Committee: 2007/381 and Clinical Trial.gov Identifier Number: NCT 00738231). In addition to its identity as a developed province of Turkey, Eskisehir is also considered to be a region with a developing rural area. As of 2006, the total population of the study region was 10,510, and the population of individuals over the age of 40 was 5,386 (51.2%) [12]. The main source of income of the population is farming. The literacy rate in the region is 95 %.

At the beginning of the study, the sampling volume (confidence interval = 95%, α = 5%, β = 20%, change rate in risk factors = 5%) was estimated to be around 500 people. The study consisted of three stages, and was completed between September 2005 and July 2007. Since it was a follow-up study, it was started with a sampling volume that was 10% larger than the calculated volume required.

### 2.2. Procedures

In the first stage of the study, 555 individuals over 40 years of age were contacted. Individuals who were literate or had at least one literate person in the household were selected through random sampling via the records of local health care units. The average age of the individuals was 57.34 ± 11.85, and the gender distribution was 46.8% male and 53.2% female.

### 2.3. Analytical Procedures

The individuals were asked about their personal information, socio-demographic characteristics, and cardiovascular disease risk factors (e.g., nutritional characteristics, smoking habits, diseases diagnosed by a physician). Throughout the study period, blood pressure, weight, and height were measured. A survey of 19 questions was implemented using a scale derived from the literature that had been adapted for Turkish nationals to measure individuals’ present knowledge on cardiovascular disease risk factors [13]. Answers to the questions were in the form of ‘yes’, ‘no’, and ‘I do not know’. The questions included on the survey related to the following: coming to terms with having a heart disease, being aware of family predisposition, advanced age as a risk factor, smoking as a risk factor, being aware of the effects of quitting smoking, being aware of hypertension as a risk factor, knowing the effect of keeping blood pressure under control, being aware of high levels of cholesterol as a risk factor, the impact of fatty food in rising cholesterol levels, being aware of the effect of good cholesterol and bad cholesterol, knowing about weight as a risk factor, being aware that regular physical activity decreases risk, knowing that risk decreases by merely working out in the gym, being aware of diabetes as a risk factor, knowing the effect of sugar consumption in diabetes knowing the cholesterol level of diabetics, being aware of the impact of weight and blood pressure control on heart diseases in diabetics, and being aware that diabetic men have higher risk compared to diabetic women. Level of knowledge was measured by assigning a value of ‘1’ for every affirmative answer.

Individuals who regularly smoke one or more cigarettes a day were considered to be ‘smokers’ [14]. With regard to nutrition, oil, fresh vegetables and fruits, red meat, and added salt consumption were also assessed. Proper nutritional behavior was defined as consuming animal fat and margarine, vegetable and fruit (at least four days per week), and red meat (at least two days per week). Additional use of salt in meals was deemed inappropriate behavior [15–18].

Physical activity levels were classified as either mild or heavy, according to literature [19]. Participant heights and weights were measured appropriately. In classifying body weight according to the Body Mass Index (BMI), individuals at 25 kg/m^2^ and above were assessed to be overweight [20].

After completing the survey, the systolic and diastolic blood pressures were measured from the left arm of participants sitting upright, using the Korotkoff Phases 1 and 5 to acquire the measurement. In accordance with World Health Organization recommendations, individuals with a 140 mmHg and above systolic blood pressure and/or with a diastolic blood pressure of 90 mmHg and above, as well as those who were previously diagnosed with hypertension and had been taking medications, were deemed hypertensive, even if their blood pressure levels were normal at that time [21].

In the second stage of the study, public training materials about cardiovascular disease were distributed. The training materials were of two types: Group I’s training material consisted of a brochure with the information we wanted the public to know about heart disease. The brochure had such titles as “Cardiovascular Diseases, let us protect our hearts, the importance of cholesterol in preventing heart diseases, watch out for blood pressure, quit smoking for your health, weight watching, nutrition, food to avoid in cardiovascular disease, an easy method: exercise and exercise control, and an appropriate body weight vs. height chart for adults” [22].

The brochure had dimensions of 21 cm × 7.5 cm and included 15 small photos demonstrating various cognitive and behavioral strategies, predominantly for hypertension, hypercholesterolemia, smoking, obesity, sedentary lifestyle, and diabetes. In the brochure, the cognitive strategies identified to combat each of the risk factors consisted of increasing the level of knowledge, warning about the risks, drawing attention to the importance of the issue, and increasing understanding the benefits of keeping the risk factors under control. Among the behavioral strategies, the brochure merely showed the path to avoid risk factors and provided information about them. The Group II training material document was a letter in the form of a prescription in which the individual was addressed by name, the risk factors established at the first stage were explained, and the suggested measures for protection from such risk factors were indicated.

The training materials were randomly distributed to the study participant groups. The list of names was systematically divided into two groups: odd-numbered individuals were given brochures only, and even-numbered individuals were given both letters and brochures. Since we could not have prevented an illustrated material to be passed by in a closed environment, it was deemed appropriate to distribute both the letter and brochure instead of just the brochure.

In the second stage of the study, individuals were contacted again 15 days following distribution of the training material, and the change in their level of knowledge was investigated. The 19 same questions which the level of knowledge questionnaire from the first survey were asked again [13]. Those who reported not having read the training materials (n = 8 (1.4%)) were excluded from the study.

**Figure 1.**
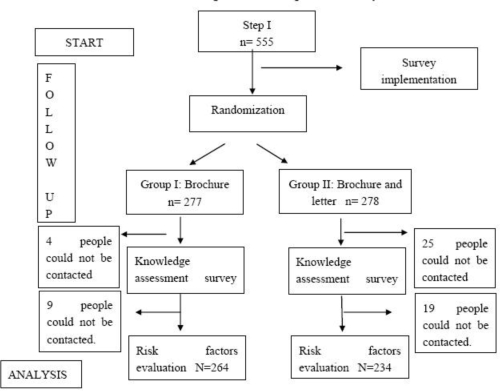
Flow diagram of the study.

The third stage took place around a year after beginning the study. The objective of the third stage was to evaluate the effectiveness of the training materials and to determine what behavioral changes had been implemented to reduce the risk factors of coronary heart diseases. The third stage of the study consisted of a survey that was administered to 498 (89.7%) people out of 555. Fifty-seven people who had participated in the earlier portion of the study could not be reached because they were either deceased (7), had emigrated (22), or were temporarily away from the district (28). No difference was observed between individuals who left during the follow-up and those who were able to complete the study in terms of age, gender, and socio-economic criteria (p > 0.05). The average age of individuals who participated in the [third stage of the] study was 57.17 ± 11.97, of which 53.2% were females and 46.2% were males. Behavioral changes in the risk factors following the training were examined through the survey. Criteria adopted in the first stage were taken as the basis for evaluating the risk factors. The flow diagram of the study is given in Figure 1.

### 2.4. Statistical Analyses

Software packs were used to evaluate the data. Pearson X^2^, t test for dependent samples, Mc-Nemar X^2^, and the t test in matched series were applied. Linear regression was used to obtain a corrected p value in the analysis of the measured values, logistic regression analysis was utilized to obtain a corrected p value in the analysis of qualitative values.

Age, gender, education level, presence of social security, marital status, overweight status, smoking behavior, sedentary lifestyle, presence of cardiovascular disease as diagnosed by a physician, diabetes, and hypertension, were taken as the independent variables affecting the individuals that could prompt desired behavioral changes in at least one of the four parameters (being overweight, smoking, high blood pressure, sedentary lifestyle). For the single variable analyses, a logistic regression model was employed with a significance level of p < 0.10.

## 3. Results

Of the 498 people participating in all three stages of the study, 60% were younger than 60, 46.2% were females, 53.8% were males, 82.5% had less than eight years of education, 87.8% were married, and 90.2% had social security. Among the 264 people that received Group I training materials and the 234 people that received Group II training materials, no difference was observed in terms of socio-demographic characteristics (age groups, gender, education level, marital status, and the receipt of social security) (p > 0.05).

Risky behaviors observed among the individuals in the study group related to the consumption of fat (29.3%), salt (15.1%), red meat (7%), and insufficient consumption of vegetables and fruits (82.7%). Physically inactive individuals comprised 20.5% of the group, 69.9% were overweight, and 30.3% were smokers. Rates for hypertension, diabetes, and diagnosis of cardiovascular disease were 46.2%, 9.4% and 19.9%, respectively.

The average score on the survey obtained before training was 9.51 ± 4.68 (min-max: 0–19), and rose to 14.01 ± 2.99 (min-max: 4–19) after training (p < 0.001). Score increases were found to be significant (p < 0.001). The score changes of individuals who were given Group I and Group II training materials were not observed to have significant differences after their adjusted for the first scores (p = 0.447).

Nutritional habits exhibited significant changes after the training, except for excessive consumption of fat. The change in nutritional habits did not show any difference between the training material groups. While the most inappropriately consumed food group was vegetables and fruits, the scarcity in red meat consumption was due to an inability to obtain this food type (Table 1).

A significant decrease was observed after the training in terms of systolic sand diastolic blood pressure among individuals in both groups. The decrease in systolic and diastolic blood pressure levels was 2 mmHg. A difference was not reported between the training materials in terms of efficiency (Table 2).

The blood pressure levels decreased in 6.5% (p = 0.10) of individuals who were given Group I training materials, and in 12.4 % (p = 0.003) of individuals who were given Group II training materials. Among those who were given Group II training materials, arterial blood pressure regulation after the training was found to have significantly increased.

Though not significant, the weights of individuals with Group II training material manifested a decrease (p = 0.090). Only individuals who were given the Group II training material had a significant drop in BMI after the training (Table 2).

The number of active smokers who were given either Group I or Group II training materials showed a significant decrease after the training (Table 3).

In the third stage of the study, 182 people (36.5%) were identified to have manifested a change in at least one of the four risky behaviors. In establishing effective variables concerning positive behavioral changes, it was not possible to identify significant effects of gender (p = 0.583), social security intake (p = 0.733), marital status (p = 0.841), cardiovascular disease diagnosis (p = 0.813), diabetes diagnosis of (p = 0.708), and smoking habits (p = 0.627).

There was no effect of the level of score change on the behavioral change (p = 0.144). Age, training period, hypertension, physical activity, and weight were included in the logistic model used to determine the independent variables affecting positive behavioral changes. It was not possible to judge the effect of education level and overweight status on the positive behavioral change. The positive impact from the training increased values 1.6 times with age over 60 years, 4.5 times in people with hypertension, and 8.7 times in people who were physically sedentary (Table 4).

## 4. Discussion

Advances in social principle theories and behavioral change theory models have determined the course of specific cognitive and behavioral processes in encouraging appropriate behavioral changes in health. Behavioral changes are second to cognitive changes in terms of which type of changes should take priority [13].

Studies on controlling cardiovascular risks are plentiful, with national media campaigns, training materials, conferences, behavior change-specific programs, and public march programs being examined. It is a known fact in many countries that public health training endeavors related to cardiovascular diseases expand the awareness and knowledge of risk factors [24–26]. In order to avoid many risk factors, it was shown that willingness to participate increases the likelihood of the individual starting the activity [22].

Apart from the fact that education efforts increase knowledge, awareness, and willingness to adopt behavior changes, it is necessary for the knowledge to be transformed into healthy behavior. There is a need for studies to find methods to stimulate this transformation. The objective of this study was to compare the two sets of training materials prepared in order to analyze the effect of the information presentation format.

It was observed that, in groups with low socio-economic and education levels that had more difficulty accessing information, the studies produced more successful results. The geographic area in which the present study was implemented is an example of such a region. The study adopted the individual interview method of community-based protection programs. First, the risk factors of the individuals were analyzed, and their awareness of the risk factors was expanded through verbal dialogue. Then, they received the training materials. Group II training materials addressed the individuals by name, explained what should be done regarding the cardiovascular risk factors unique to each person, and were designed in the fashion of a prescription.

As is the case in many studies, our data showed no significant problems with presenting the information [27–29]. The survey used examined the participant’s level of knowledge about the risk factors, and the number of correct answers was quite satisfactory after the training. A difference was not observed between the training materials in terms of their ability to increase the individual’s level of knowledge.

Today, it is known that unhealthy nutrition has a negative effect on cardiovascular disease. Consumption of food with high saturated fat levels, high levels of salt, large amounts of carbohydrates, and excessive consumption of red meat are among the examples of unhealthy nutritional behaviors. Since regular consumption of food rich in fiber such as fruits, vegetables and grains are known to have a preventive effect, insufficient intake of these products constitutes a risk for cardiovascular disease.

In studies analyzing the effects of diet and implementing an aggressive limitation of fat intake, a roughly 30 to 60 percent decrease in deaths was observed [30]. However, the desired significant change regarding fat use was not observed though a positive change in overall nutritional habits was obtained after the training. In fact, the type of fat used by a given household is an established habit and behavior pattern, and is also a function of the economic status of the family. Solid fats like margarine are cheap, whereas olive oil is expensive.

In a study conducted by Cutler *et al*. on limiting salt intake, it was reported that a 50% decrease in the salt intake could probably result in a significant drop in high blood pressure [31]. In the study, individuals who consumed appropriate amounts of salt manifested a 7.25% decrease in blood pressure; this drop was not due to salt added to meals during cooking, but rather to the amount of salt added to meals after cooking. Limiting salt intake is not a practice generally adopted by the public, except hypertension patients.

The results obtained concerning red meat consumption were not different from the pre-training results. In the group with low red meat consumption (due to economic constraints), only 3.5% of participants’ scores related to appropriate meat consumption were affected.

Although a significant increase of 23.35% was obtained for vegetable and fruit consumption after the training, the level of vegetable and fruit consumption in the study group was still lower than desirable.

According to Framingham Coronary Profile Risk Factor Scoring, cardiovascular disease risk was reported to have shown a 15% drop due to an increase in participant physical activity [32]. Also, it was reported in a TEKHARF Study (Turkish Adults Risk Factor Study) carried out in Turkey that, through regular and appropriate exercise, a 23 percent decrease in deaths due to cardiovascular diseases was seen [33]. Similarly, in a study conducted to determine the physical activity levels of women in Croatia, the physical activity level among housewives below the age of 50 was reported to be low, but the activity level increased with patient participation in appropriate supportive programs [34].

Although it is a known fact that physical activities have positive effects on health, it is difficult to bring about a change in behavior that encourages increasing the level of physical activity of individuals. Although working out might be known to improve health, only a very small portion of the adult population, most of which are of a higher education level, participate in such activities [35,36].

A change in one’s level of physical activity would impact most of the cardiovascular risk factors and create significant changes in the blood lipid profile, hypertension, and body composition. The most important reason why we were not able to obtain the expected level of success in terms of physical activity in our study was the lack of locations to carry out outdoor activities within the geographic region where our study was conducted. From a cultural point of view, a woman living in a rural area does not leave the house very often, nor does she have a habit of walking regularly (apart from her housework). Also, men in rural areas tend to spend their time gathering in coffee houses, especially in the winter. It would thus be appropriate to develop social support policies in addition to providing information on an individual basis concerning the need to increase physical activity.

A significant decrease (2.1%) in the number of individuals with a BMI > 25 was not observed after the training. A 2.1% drop was observed in the overweight group after the training. It is clear that the dietary habits of people can change, but a decrease in BMI is only possible through systematic, long-term health education supplemented with behavioral change strategies [23]. In situations where we were not able to inspire an increase the physical activity level, we should have expected a change in BMI as a result of changes in the nutritional habits. A primary prevention study on obesity is complicated by variations in genetic predisposition, and many risk factors known for cardiovascular disease have a wider-reach than strictly behavior [23].

One of the most positive effects, in terms of lifestyle, manifested itself in the participants’ systolic and diastolic blood pressures. Around a 2 mm Hg drop in systolic and diastolic blood pressure was observed after the training. The rate of regulated blood pressure among the individuals who were given Group II training materials was observed to have a significant increase. In our region, where the number of people with ideal blood pressure levels is quite high, the fact that successful results were obtained with minimal effort should prove informative for future studies. As a result of a research program by Kisioglu *et al*. that incorporated training on how to better control high blood pressure among middle-aged women of low socio-economic level in Isparta, hypertension prevalence was reported to decrease [37].

Smoking is an important public health issue in Turkey and in the rest of the world. Although the number of smokers in developed countries has been decreasing since the seventies, it has shown an increase in developing countries. As a result of training programs and other measures taken against smoking, 36 million people in the USA, 8 million people in France, and 1 million people in Switzerland have quit smoking [38].

According to the results obtained from the TUMAR (Multi-centered Turkish Myocardial Infarction Study) in Turkey, the number of smokers was reported to be 17 million [33]. In a EURO-ASPIRE III study, the population of Turkey was reported to have the highest probability of individuals below the age of 50 having myocardial infarction (relative to 22 European countries). The prevalence of smoking plays an important role in this finding [39]. A 6.3% decrease in active smoking occurred in the study. Considering that quitting smoking is a difficult and lengthy process, it is quite an achievement that this outcome was obtained in such a short period of time.

Among the 182 people (36.5%) manifesting a change in at least one of four risky behaviors (namely losing weight, quitting smoking, regulating blood pressure, and increasing physical activity), individuals who were above the age of 60 years, diagnosed with hypertension, and/or, leading sedentary lifestyles were observed to be more positively affected by the training.

However, it is more important to have younger groups adapt healthy lifestyles it takes a long time for disease to develop. An immediate decrease in the amount of contact with risk factors would reduce the risk of cardiovascular disease. The fact that behavioral changes in individuals with sedentary lifestyles occurred at rates 8.7 times higher than those of physically active individuals, despite our inability to achieve the desired training success in the group of people with sedentary lifestyles, might be due to the fact that individuals were unable to adapt to changing such risk factors as quitting smoking and losing weight. The number of behavioral changes that occurred in individuals diagnosed with hypertension suggests a future training group that could yield immediate results.

Although there was no difference between the Group I and Group II training materials for many parameters of the study, the Group II training material proved to be superior for controlling blood pressure. We thus deem use of this material to be a method for implementing a salutogenic approach, whereby a human assumes a healthy lifestyle through the best utilization of his unique opportunities. The aim of preparing the Group II training material was to enable the individual to see his or her risks as unique, and to proceed with life by adapting personal behaviors. The Group II training material document was the same brochure by Group I and a letter in the form of a prescription. This letter was a more remarkable because it has appealed to people. On the other hand Turkish people like to get a prescription from a doctor.

## 5. Conclusions

One of the most significant disadvantages of community-based training programs is the low success rate. As a matter of fact, it might be difficult to truthfully determine the effect of changing individual risk factors on health outcomes. A drop in the risks for cardiovascular disease could be more attributable to decreases in blood cholesterol level, blood pressure, and BMI. When more than one change in behaviors is observed in the same individual, a multiplied decrease would occur in morbidity and mortality [24]. Because of the prevalence of unhealthy behaviors in risk factors in all societies, small changes could produce significant health effects.

This study demonstrated that while an individual’s knowledge about certain risk factors might increase, one can expect only limited success with inspiring lifestyle changes (aside: we recognize that the training implemented here was little more than a crude scaling tool). However, we can conclude that social programs oriented towards individual behavioral changes assume an important role in decreasing cardiovascular risk factors.

## Figures and Tables

**Figure 2.**
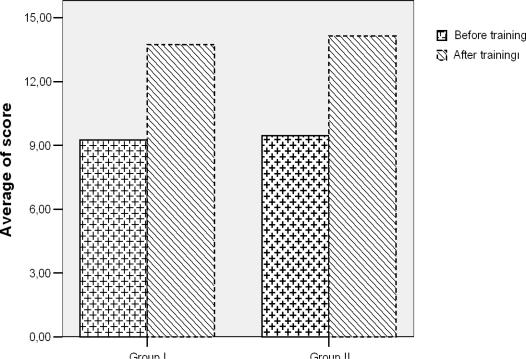
Distribution of average knowledge scores to for each set of training materials before and after the training.

**Table 1.** Before and after training change characteristics of risky nutritional habits.

Variables	Training material		Analysis of Change (P)*		ΔGroup I/ΔGroup II P**
	Group I Group II (n = 264) (n = 234)		Group I	Group II	
Inappropriate fat consumption					
Starting value	80 (30.3)	66 (28.2)			0,964
After the training	72 (27.3)	53 (22.6)	0.40	0.105	
Change	−3.0	−5.6			
Inappropriate salt consumption					
Starting value	41 (15.5)	34 (14.5)	<0.001	<0.001	0.728
After the training	23 (8.7)	16 (6.8)			
Change	−6.8	−7.7			
Inappropriate red meat consumption					
Starting value	27 (10.2)	12 (5.1)	<0.001	<0.001	0.105
After the training	14 (5.3)	7 (3.0)			
Change	−4.9	−2.1			
Inappropriate vegetable/fruit consumption					
Starting value	215 (81.4)	197 (84.2)	<0.001	<0.001	0.897
After the training	156 (59.1)	140 (59.8)			
Change	−22.3	−24.4			

*: The Mc-Nemar X^2^ test was utilized to analyze the change that occurred after the training.

**: The difference in the change between the training materials was adjusted through logistic regression based on age and gender.

**Table 2.** Before and after training change characteristics of scalable cardiovascular risks.

Variables	Training material		Analysis of Change (P)*		ΔGroup I/ΔGroup IIP**
	Group I (n = 264)	Group II (n = 234)	Group I	Group II	
SBP mmHg					
Starting value	133.55 ± 21.64	134.67 ± 19.39	0.007	0.007	0.968
After the training	131.93 ± 18.30	132.20 ± 19.93			
Change	−1.62	−2.47			
DBP					
Starting value	84.13 ± 12.58	85.06 ± 13.15	0.001	0.005	0.960
After the training	82.65 ± 11.33	83.59 ± 13.56			
Change	−1.48	−1.47			
Weight (kg)					
Starting value	73.12 ± 12.99	74.56 ± 11.88	0.678	0.090	0.457
After the training	72.89 ± 12.96	73.59 ± 12.54			
Change	−0.23	−0.97			
BMI (kg/m^2^)					
Starting value	27.60 ± 4.81	28.27 ± 4.81	0.427	0.010	0.184
After the training	27.55 ± 4.96	27.83 ± 5.11			
Change	−0.05	−0.44			

*: In the analysis of the change that occurred after the training, the p value was adjusted based on pre-training values and calculated with linear regression.

**: p value of the difference between the training materials adjusted using pre-training values and calculated with linear regression. SBP: Systolic blood pressure, DBP: Diastolic blood pressure, BMI: Body Mass Index.

**Table 3.** Before and after training changes in cardiovascular risk factors.

Variables	Training material		Analysis of Change (P)*		ΔGroup I/ΔGroup II P**
	Group I (n = 264)	Group II (n = 234)	Group I	Group II	
Sedentary lifestyle rate					
Starting value	61 (23.1)	41 (17.5)	0.182	0.576	0.765
After the training	49 (18.6)	36 (15.4)			
Change	−4.5	−2.1			
Rate of regulated TA					
Starting value	114 (43.2)	119 (50.9)	0.100	0.003	0.549
After the training	97(36.7)	90 (38.5)			
Change	−6.5	−12.4			
Rate of BMI>25					
Starting value	180 (68.2)	168 (71.8)	0.892	0.268	0.806
After the training	178 (67.4)	160 (68.4)			
Change	−0.8	−3.4			
Active smoker rate					
Starting value	82 (31.1)	71 (30.4)	0.004	0.002	0.624
After the training	69 (26.2)	53 (22.7)			
Change	−4.9	−7.7			

*: The Mc-Nemar X^2^ test was utilized to analyze the change occurred after the training.

**: Difference in change between the training materials was adjusted using logistic regression based on age and gender.

**Table 4.** Logistic model showing the effect of independent variables on behavioral changes.

Variables	Rate of people showing changes (%)	OR	%95CI	P
Age				
Below 60	28.4	1		
60 and above	48.7	1.611	1.039–2.498	0.033
Period of education				
8 years or less	37.3	1		
8 and above	32.9	1.134	0.634–2.031	0.671
Hypertension				
No diagnosis	22.8	1		
With diagnosis	52.6	4.569	2.921–7.147	0.000
Physical activity				
Active	27.0	1		
Sedentary	73.5	8.778	5.055–15.244	0.000
BMI>25				
Non-overweight	34.0	1		
Overweight	37.6	1.154	0.714–1.865	0.559
